# {2,6-Bis[(diphenyl­phosphan­yl)­oxy]phen­yl-κ^3^ *P*,*C* ^1^,*P*′}iodidonickel(II)

**DOI:** 10.1107/S1600536811008828

**Published:** 2011-03-12

**Authors:** Abderrahmen Salah, Davit Zargarian

**Affiliations:** a Département de Chimie, Université de Montréal, CP 6128, Succ. Centre-ville, Montréal, Québec, Canada H3C 3J7

## Abstract

In the title complex, [Ni(C_30_H_23_O_2_P_2_)I], the divalent Ni atom is coordinated by two P atoms and one C atom from the 1,3-bis­[(diphenyl­phosphan­yl)­oxy]benzene ligand; the distorted square-planar geometry is completed by an iodide ligand. The largest distortions from ideal square-planar geometry are reflected in the P—Ni—P angle of 164.20 (2)° and the P—Ni—C angles of 82.09 (6) and 82.11 (6)°. The rather short Ni—C bond length [1.890 (2) Å] is anti­cipated in light of the much stronger *trans* influence of the aryl moiety compared to the iodide ligand. The P-bound phenyl rings adopt different orientations to minimize steric repulsion among themselves.

## Related literature

For general background to pincer complexes and their applications, see: Leis *et al.* (2008 ▶); Dijkstra *et al.* (2001 ▶); Naghipour *et al.* (2007 ▶); van der Boom & Milstein (2003 ▶); Nishiyama (2007 ▶).
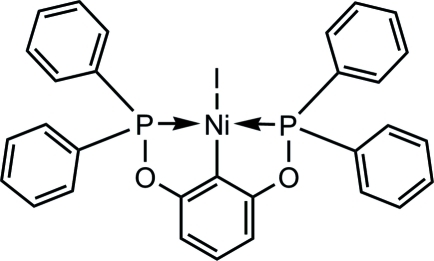


## Experimental

### 

#### Crystal data

[Ni(C_30_H_23_O_2_P_2_)I]
*M* *_r_* = 663.03
Monoclinic, 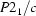

*a* = 16.4446 (3) Å
*b* = 10.8531 (2) Å
*c* = 17.3131 (3) Å
β = 120.429 (1)°
*V* = 2664.33 (8) Å^3^
*Z* = 4
Cu *K*α radiation
μ = 11.49 mm^−1^
*T* = 150 K
0.18 × 0.10 × 0.09 mm

#### Data collection

Bruker SMART 6000 diffractometer
Absorption correction: multi-scan (*SADABS*; Sheldrick, 1996 ▶) *T*_min_ = 0.198, *T*_max_ = 0.356
35047 measured reflections
5259 independent reflections
5142 reflections with *I* > 2σ(*I*)
*R*_int_ = 0.034

#### Refinement

*R*[*F* ^2^ > 2σ(*F* ^2^)] = 0.026
*wR*(*F* ^2^) = 0.068
*S* = 1.07
5259 reflections
326 parameters
H-atom parameters constrained
Δρ_max_ = 0.75 e Å^−3^
Δρ_min_ = −0.78 e Å^−3^

### 

Data collection: *APEX2* (Bruker, 2007 ▶); cell refinement: *SAINT* (Bruker, 2007 ▶); data reduction: *SAINT*; program(s) used to solve structure: *SHELXTL* (Sheldrick, 2008 ▶); program(s) used to refine structure: *SHELXTL*; molecular graphics: *SHELXTL*; software used to prepare material for publication: *UdMX* (Maris, 2004 ▶) and *publCIF* (Westrip, 2010 ▶).

## Supplementary Material

Crystal structure: contains datablocks I, global. DOI: 10.1107/S1600536811008828/nk2088sup1.cif

Structure factors: contains datablocks I. DOI: 10.1107/S1600536811008828/nk2088Isup2.hkl

Additional supplementary materials:  crystallographic information; 3D view; checkCIF report

## Figures and Tables

**Table:** 

I1—Ni1	2.4976 (3)
Ni1—P1	2.1553 (5)
Ni1—P2	2.1601 (5)

**Table:** 

C1—Ni1—I1	178.93 (6)
P1—Ni1—I1	97.239 (17)
P2—Ni1—I1	98.556 (16)

**Fig. 1.**
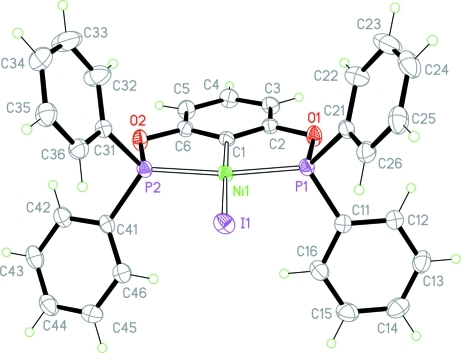
The molecular structure of the title compound, showing 50% probability displacement ellipsoids.

## supplementary crystallographic information

### Comment

Recently, much attention has been paid to the chemistry of pincer complexes (Leis *et al.* 2008; Dijkstra *et al.* 2001; Naghipour *et al.* 2007; van der Boom *et al.* 2003; Nishiyama 2007). These compounds have found applications as promising materials and highly versatile catalysts. Here we report the crystal structure and the synthesis of{*m*-(Ph_2_PO)_2_ C_6_H_3_}NiI. The formation of the title complex was serendipitous in that the original goal of the synthesis was to prepare the corresponding methyl derivative {*m*-(Ph_2_PO)_2_C_6_H_3_}Ni(CH_3_). To our surprise, reaction of the bromo precursor with the Grignard reagent MeMgI gave instead the iodo derivative. It appears that the target methyl complex is not sufficiently stable, undergoing a salt metathesis with MgX_2_ (*X*= Br or I) to furnish the iodo derivative. As shown in Fig. 1, the Ni(II) center in the title complex exists in the center of a square plane defined by the donor atoms P1 and P2, the iodide ligand, and the carbon atom of the aromatic moiety of the pincer ligand. Despite the rigid meridional coordination of the tridentate pincer-type ligand, a slight distortion is evident in the solid state of this complex from the P—Ni—P angles of 82.09 (6) and 82.11 (6)°; such distortions are commonly found in this family of Ni(II) pincer complexes (van der Boom *et al.* 2003).

### Experimental

Transfer of MeMgI (0.12 ml of a 3.0 *M* solution in THF, 0.24 mmol) to a stirred solution of {*m*-(Ph2PO)2 C6H3}NiBr(50 mg, 0.08 mmol) in dry and degassed toluene (1.5 ml) caused an immediate color change from deep yellow to a red orange. The resulting mixture was stirred under an inert atmosphere of nitrogen for 15 min and was then filtered through cellulose. Evaporation of the solvent gave an orange solid. Single crystals suitable for X-ray diffraction studies were grown by slowly diffusing hexane into a saturated toluene solution.

### Refinement

All hydrogen atoms were positioned geometrically and refined as riding, with C—H = 0.93 Å, and *U*_iso_(H) = 1.2 *U*_eq_(C).

### Crystal data

**Table:**

[Ni(C_30_H_23_O_2_P_2_)I]	*F*(000) = 1320
*M**_r_* = 663.03	*D*_x_ = 1.653 Mg m^−^^3^
Monoclinic, *P*2_1_/*c*	Cu *K*α radiation, λ = 1.54178 Å
Hall symbol: -P 2ybc	Cell parameters from 24450 reflections
*a* = 16.4446 (3) Å	θ = 5.1–72.4°
*b* = 10.8531 (2) Å	µ = 11.49 mm^−^^1^
*c* = 17.3131 (3) Å	*T* = 150 K
β = 120.429 (1)°	Block, yellow
*V* = 2664.33 (8) Å^3^	0.18 × 0.10 × 0.09 mm
*Z* = 4	

### Data collection

**Table:**

Bruker SMART 6000 diffractometer	5259 independent reflections
Radiation source: X-ray Sealed Tube	5142 reflections with *I* > 2σ(*I*)
graphite	*R*_int_ = 0.034
Detector resolution: 5.5 pixels mm^-1^	θ_max_ = 72.4°, θ_min_ = 3.1°
ω scans	*h* = −20→20
Absorption correction: multi-scan (*SADABS*; Sheldrick, 1996)	*k* = −13→13
*T*_min_ = 0.198, *T*_max_ = 0.356	*l* = −21→21
35047 measured reflections	

### Refinement

**Table:**

Refinement on *F*^2^	Secondary atom site location: difference Fourier map
Least-squares matrix: full	Hydrogen site location: inferred from neighbouring sites
*R*[*F*^2^ > 2σ(*F*^2^)] = 0.026	H-atom parameters constrained
*wR*(*F*^2^) = 0.068	*w* = 1/[σ^2^(*F*_o_^2^) + (0.0502*P*)^2^ + 0.5043*P*] where *P* = (*F*_o_^2^ + 2*F*_c_^2^)/3
*S* = 1.07	(Δ/σ)_max_ = 0.007
5259 reflections	Δρ_max_ = 0.75 e Å^−^^3^
326 parameters	Δρ_min_ = −0.78 e Å^−^^3^
0 restraints	Extinction correction: *SHELXTL* (Sheldrick, 2008), Fc^*^=kFc[1+0.001xFc^2^λ^3^/sin(2θ)]^-1/4^
Primary atom site location: structure-invariant direct methods	Extinction coefficient: 0.00190 (6)

### Special details

**Table:**

Experimental. X-ray crystallographic data for I were collected from a single-crystal sample, which was mounted on a loop fiber. Data were collected using a Bruker Platform diffractometer, equipped with a Bruker *SMART* 2 K Charged-Coupled Device (CCD) Area Detector using the program *SMART* and normal focus sealed tube source graphite monochromated Cu—Kα radiation. The crystal-to-detector distance was 4.908 cm, and the data collection was carried out in 512 *x* 512 pixel mode, utilizing 4 *x* 4 pixel binning. The initial unit-cell parameters were determined by a least-squares fit of the angular setting of strong reflections, collected by a 9.0 degree scan in 30 frames over four different parts of the reciprocal space (120 frames total). One complete sphere of data was collected, to better than 0.8Å resolution. Upon completion of the data collection, the first 101 frames were recollected in order to improve the decay correction analysis.
Geometry. All e.s.d.'s (except the e.s.d. in the dihedral angle between two l.s. planes) are estimated using the full covariance matrix. The cell e.s.d.'s are taken into account individually in the estimation of e.s.d.'s in distances, angles and torsion angles; correlations between e.s.d.'s in cell parameters are only used when they are defined by crystal symmetry. An approximate (isotropic) treatment of cell e.s.d.'s is used for estimating e.s.d.'s involving l.s. planes.
Refinement. Refinement of *F*^2^ against ALL reflections. The weighted *R*-factor *wR* and goodness of fit *S* are based on *F*^2^, conventional *R*-factors *R* are based on *F*, with *F* set to zero for negative *F*^2^. The threshold expression of *F*^2^ > σ(*F*^2^) is used only for calculating *R*-factors(gt) *etc*. and is not relevant to the choice of reflections for refinement. *R*-factors based on *F*^2^ are statistically about twice as large as those based on *F*, and *R*- factors based on ALL data will be even larger.

### Fractional atomic coordinates and isotropic or equivalent isotropic displacement parameters (Å^2^)

**Table:**

	*x*	*y*	*z*	*U*_iso_*/*U*_eq_	
I1	0.214929 (9)	0.790060 (11)	0.756156 (8)	0.02460 (7)	
Ni1	0.23502 (2)	0.56348 (3)	0.74669 (2)	0.01757 (9)	
P1	0.10949 (3)	0.50611 (4)	0.74657 (3)	0.01912 (11)	
P2	0.36537 (3)	0.56701 (4)	0.74486 (3)	0.01912 (11)	
O1	0.10883 (9)	0.35423 (13)	0.74495 (10)	0.0252 (3)	
O2	0.39191 (10)	0.42230 (12)	0.73844 (10)	0.0254 (3)	
C1	0.24998 (13)	0.39159 (18)	0.74142 (11)	0.0192 (4)	
C2	0.18453 (14)	0.30761 (18)	0.73952 (13)	0.0204 (4)	
C3	0.19118 (15)	0.18122 (19)	0.73052 (14)	0.0257 (4)	
H3	0.1470	0.1274	0.7302	0.031*	
C4	0.26582 (15)	0.13764 (19)	0.72202 (15)	0.0291 (4)	
H4	0.2712	0.0534	0.7156	0.035*	
C5	0.33263 (16)	0.21712 (18)	0.72286 (16)	0.0278 (5)	
H5	0.3815	0.1876	0.7157	0.033*	
C6	0.32412 (13)	0.34153 (18)	0.73477 (13)	0.0214 (4)	
C11	−0.00440 (13)	0.54428 (17)	0.65138 (12)	0.0217 (4)	
C12	−0.08627 (14)	0.5049 (2)	0.64934 (14)	0.0269 (4)	
H12	−0.0819	0.4629	0.6981	0.032*	
C13	−0.17438 (15)	0.5282 (2)	0.57474 (15)	0.0335 (5)	
H13	−0.2288	0.5011	0.5733	0.040*	
C14	−0.18063 (16)	0.5921 (2)	0.50231 (14)	0.0342 (5)	
H14	−0.2394	0.6089	0.4526	0.041*	
C15	−0.09947 (16)	0.6307 (2)	0.50407 (14)	0.0345 (5)	
H15	−0.1041	0.6727	0.4552	0.041*	
C16	−0.01117 (15)	0.6074 (2)	0.57815 (13)	0.0292 (4)	
H16	0.0431	0.6337	0.5789	0.035*	
C21	0.09797 (13)	0.53687 (19)	0.84293 (12)	0.0226 (4)	
C22	0.12540 (17)	0.4497 (2)	0.91095 (14)	0.0345 (5)	
H22	0.1469	0.3727	0.9057	0.041*	
C23	0.1204 (2)	0.4787 (3)	0.98674 (16)	0.0452 (6)	
H23	0.1385	0.4206	1.0321	0.054*	
C24	0.08858 (17)	0.5934 (3)	0.99508 (14)	0.0401 (6)	
H24	0.0854	0.6121	1.0459	0.048*	
C25	0.06161 (16)	0.6797 (2)	0.92806 (16)	0.0345 (5)	
H25	0.0405	0.7568	0.9338	0.041*	
C26	0.06594 (14)	0.6518 (2)	0.85174 (14)	0.0273 (4)	
H26	0.0474	0.7101	0.8065	0.033*	
C31	0.47066 (13)	0.61934 (18)	0.84480 (12)	0.0226 (4)	
C32	0.52646 (18)	0.5369 (2)	0.91276 (17)	0.0422 (6)	
H32	0.5122	0.4533	0.9053	0.051*	
C33	0.60360 (19)	0.5792 (3)	0.99183 (17)	0.0501 (7)	
H33	0.6408	0.5234	1.0369	0.060*	
C34	0.62544 (17)	0.7026 (2)	1.00406 (17)	0.0372 (5)	
H34	0.6767	0.7304	1.0574	0.045*	
C35	0.57086 (17)	0.7847 (2)	0.93671 (16)	0.0310 (5)	
H35	0.5859	0.8682	0.9444	0.037*	
C36	0.49354 (14)	0.7437 (2)	0.85732 (14)	0.0271 (4)	
H36	0.4570	0.7999	0.8124	0.033*	
C41	0.37335 (13)	0.63478 (18)	0.65338 (12)	0.0211 (4)	
C42	0.44862 (15)	0.5993 (2)	0.64218 (14)	0.0287 (4)	
H42	0.4937	0.5443	0.6822	0.034*	
C43	0.45609 (16)	0.6459 (2)	0.57138 (15)	0.0333 (5)	
H43	0.5060	0.6221	0.5639	0.040*	
C44	0.38928 (17)	0.7279 (2)	0.51212 (15)	0.0334 (5)	
H44	0.3946	0.7596	0.4650	0.040*	
C45	0.31474 (17)	0.7628 (3)	0.52257 (16)	0.0378 (5)	
H45	0.2700	0.8179	0.4824	0.045*	
C46	0.30611 (16)	0.7159 (2)	0.59306 (16)	0.0311 (5)	
H46	0.2554	0.7389	0.5995	0.037*	

### Atomic displacement parameters (Å^2^)

**Table:**

	*U*^11^	*U*^22^	*U*^33^	*U*^12^	*U*^13^	*U*^23^
I1	0.03220 (10)	0.01565 (9)	0.03019 (10)	0.00088 (4)	0.01890 (7)	−0.00007 (4)
Ni1	0.01882 (16)	0.01435 (16)	0.02162 (16)	−0.00080 (11)	0.01176 (13)	0.00051 (11)
P1	0.0198 (2)	0.0171 (2)	0.0232 (2)	−0.00065 (16)	0.01296 (18)	0.00082 (17)
P2	0.0197 (2)	0.0161 (2)	0.0239 (2)	−0.00070 (16)	0.01278 (18)	0.00062 (17)
O1	0.0243 (7)	0.0182 (6)	0.0383 (8)	−0.0022 (5)	0.0198 (6)	0.0004 (6)
O2	0.0234 (7)	0.0175 (7)	0.0417 (8)	−0.0002 (5)	0.0211 (6)	0.0003 (6)
C1	0.0209 (8)	0.0176 (9)	0.0187 (8)	−0.0007 (7)	0.0097 (7)	0.0011 (7)
C2	0.0219 (9)	0.0171 (8)	0.0228 (9)	0.0014 (7)	0.0118 (7)	0.0017 (7)
C3	0.0296 (10)	0.0161 (9)	0.0302 (10)	−0.0044 (8)	0.0144 (8)	0.0015 (8)
C4	0.0344 (10)	0.0134 (9)	0.0401 (11)	0.0001 (8)	0.0192 (9)	0.0011 (8)
C5	0.0293 (11)	0.0198 (10)	0.0380 (12)	0.0038 (7)	0.0197 (10)	0.0011 (8)
C6	0.0225 (9)	0.0168 (9)	0.0254 (9)	−0.0013 (7)	0.0125 (7)	0.0016 (7)
C11	0.0227 (9)	0.0196 (9)	0.0238 (9)	−0.0003 (7)	0.0124 (7)	−0.0026 (7)
C12	0.0249 (10)	0.0289 (10)	0.0282 (10)	0.0000 (8)	0.0145 (8)	0.0022 (8)
C13	0.0232 (10)	0.0415 (12)	0.0342 (11)	−0.0032 (9)	0.0135 (9)	−0.0029 (10)
C14	0.0291 (10)	0.0389 (12)	0.0256 (10)	0.0038 (9)	0.0072 (8)	−0.0020 (9)
C15	0.0369 (11)	0.0404 (12)	0.0230 (10)	−0.0017 (10)	0.0128 (9)	0.0037 (9)
C16	0.0311 (10)	0.0332 (11)	0.0260 (9)	−0.0055 (9)	0.0163 (8)	−0.0005 (9)
C21	0.0205 (9)	0.0272 (10)	0.0223 (9)	−0.0027 (7)	0.0125 (7)	0.0003 (8)
C22	0.0413 (12)	0.0316 (11)	0.0300 (11)	−0.0001 (9)	0.0177 (9)	0.0052 (9)
C23	0.0555 (15)	0.0526 (15)	0.0276 (11)	−0.0079 (13)	0.0211 (11)	0.0087 (11)
C24	0.0401 (12)	0.0596 (16)	0.0271 (10)	−0.0152 (11)	0.0219 (9)	−0.0107 (11)
C25	0.0299 (11)	0.0447 (12)	0.0345 (11)	−0.0043 (10)	0.0205 (9)	−0.0110 (10)
C26	0.0253 (9)	0.0320 (11)	0.0269 (10)	0.0015 (8)	0.0148 (8)	−0.0003 (8)
C31	0.0210 (8)	0.0245 (9)	0.0240 (9)	0.0001 (7)	0.0128 (7)	0.0020 (8)
C32	0.0398 (13)	0.0273 (11)	0.0401 (13)	−0.0020 (10)	0.0060 (10)	0.0076 (10)
C33	0.0427 (14)	0.0417 (15)	0.0380 (13)	−0.0005 (11)	−0.0002 (11)	0.0131 (11)
C34	0.0274 (11)	0.0459 (15)	0.0294 (11)	−0.0047 (9)	0.0078 (9)	−0.0019 (9)
C35	0.0299 (11)	0.0304 (12)	0.0320 (11)	−0.0054 (8)	0.0150 (9)	−0.0044 (8)
C36	0.0252 (10)	0.0257 (10)	0.0289 (10)	−0.0005 (8)	0.0126 (8)	0.0014 (9)
C41	0.0231 (9)	0.0200 (9)	0.0231 (9)	−0.0054 (7)	0.0137 (7)	−0.0025 (7)
C42	0.0306 (10)	0.0277 (10)	0.0337 (10)	−0.0003 (8)	0.0205 (9)	0.0007 (9)
C43	0.0362 (11)	0.0383 (12)	0.0373 (11)	−0.0046 (9)	0.0274 (10)	−0.0034 (10)
C44	0.0372 (12)	0.0413 (12)	0.0252 (10)	−0.0112 (10)	0.0184 (9)	−0.0015 (9)
C45	0.0356 (12)	0.0468 (13)	0.0314 (11)	0.0053 (11)	0.0172 (10)	0.0146 (10)
C46	0.0277 (11)	0.0387 (13)	0.0313 (11)	0.0026 (8)	0.0181 (9)	0.0057 (9)

### Geometric parameters (Å, °)

**Table:**

I1—Ni1	2.4976 (3)	C21—C22	1.395 (3)
Ni1—C1	1.890 (2)	C22—C23	1.392 (3)
Ni1—P1	2.1553 (5)	C22—H22	0.9300
Ni1—P2	2.1601 (5)	C23—C24	1.386 (4)
P1—O1	1.6486 (15)	C23—H23	0.9300
P1—C21	1.8028 (19)	C24—C25	1.378 (4)
P1—C11	1.8080 (19)	C24—H24	0.9300
P2—O2	1.6488 (14)	C25—C26	1.392 (3)
P2—C41	1.8101 (19)	C25—H25	0.9300
P2—C31	1.811 (2)	C26—H26	0.9300
O1—C2	1.390 (2)	C31—C36	1.389 (3)
O2—C6	1.394 (2)	C31—C32	1.391 (3)
C1—C6	1.392 (3)	C32—C33	1.391 (3)
C1—C2	1.398 (3)	C32—H32	0.9300
C2—C3	1.391 (3)	C33—C34	1.375 (4)
C3—C4	1.391 (3)	C33—H33	0.9300
C3—H3	0.9300	C34—C35	1.379 (3)
C4—C5	1.391 (3)	C34—H34	0.9300
C4—H4	0.9300	C35—C36	1.391 (3)
C5—C6	1.384 (3)	C35—H35	0.9300
C5—H5	0.9300	C36—H36	0.9300
C11—C16	1.395 (3)	C41—C46	1.385 (3)
C11—C12	1.396 (3)	C41—C42	1.401 (3)
C12—C13	1.391 (3)	C42—C43	1.388 (3)
C12—H12	0.9300	C42—H42	0.9300
C13—C14	1.391 (3)	C43—C44	1.381 (3)
C13—H13	0.9300	C43—H43	0.9300
C14—C15	1.385 (3)	C44—C45	1.380 (4)
C14—H14	0.9300	C44—H44	0.9300
C15—C16	1.390 (3)	C45—C46	1.394 (3)
C15—H15	0.9300	C45—H45	0.9300
C16—H16	0.9300	C46—H46	0.9300
C21—C26	1.392 (3)		
			
C1—Ni1—P1	82.11 (6)	C11—C16—H16	120.2
C1—Ni1—P2	82.09 (6)	C26—C21—C22	119.56 (19)
P1—Ni1—P2	164.20 (2)	C26—C21—P1	119.36 (15)
C1—Ni1—I1	178.93 (6)	C22—C21—P1	120.99 (17)
P1—Ni1—I1	97.239 (17)	C23—C22—C21	119.6 (2)
P2—Ni1—I1	98.556 (16)	C23—C22—H22	120.2
O1—P1—C21	101.43 (9)	C21—C22—H22	120.2
O1—P1—C11	102.63 (8)	C24—C23—C22	120.4 (2)
C21—P1—C11	104.79 (9)	C24—C23—H23	119.8
O1—P1—Ni1	106.67 (5)	C22—C23—H23	119.8
C21—P1—Ni1	119.68 (6)	C25—C24—C23	120.0 (2)
C11—P1—Ni1	118.96 (7)	C25—C24—H24	120.0
O2—P2—C41	100.90 (8)	C23—C24—H24	120.0
O2—P2—C31	101.86 (8)	C24—C25—C26	120.1 (2)
C41—P2—C31	104.52 (9)	C24—C25—H25	120.0
O2—P2—Ni1	106.42 (5)	C26—C25—H25	120.0
C41—P2—Ni1	122.15 (7)	C21—C26—C25	120.2 (2)
C31—P2—Ni1	117.85 (6)	C21—C26—H26	119.9
C2—O1—P1	111.50 (12)	C25—C26—H26	119.9
C6—O2—P2	111.67 (12)	C36—C31—C32	118.88 (19)
C6—C1—C2	116.09 (18)	C36—C31—P2	120.42 (15)
C6—C1—Ni1	122.02 (15)	C32—C31—P2	120.56 (16)
C2—C1—Ni1	121.82 (15)	C33—C32—C31	120.2 (2)
O1—C2—C3	119.35 (18)	C33—C32—H32	119.9
O1—C2—C1	117.79 (17)	C31—C32—H32	119.9
C3—C2—C1	122.84 (19)	C34—C33—C32	120.6 (2)
C2—C3—C4	118.08 (19)	C34—C33—H33	119.7
C2—C3—H3	121.0	C32—C33—H33	119.7
C4—C3—H3	121.0	C33—C34—C35	119.5 (2)
C3—C4—C5	121.50 (19)	C33—C34—H34	120.2
C3—C4—H4	119.2	C35—C34—H34	120.2
C5—C4—H4	119.2	C34—C35—C36	120.4 (2)
C6—C5—C4	117.9 (2)	C34—C35—H35	119.8
C6—C5—H5	121.1	C36—C35—H35	119.8
C4—C5—H5	121.1	C31—C36—C35	120.34 (19)
C5—C6—O2	118.79 (17)	C31—C36—H36	119.8
C5—C6—C1	123.51 (19)	C35—C36—H36	119.8
O2—C6—C1	117.68 (18)	C46—C41—C42	119.63 (19)
C16—C11—C12	119.70 (18)	C46—C41—P2	122.28 (15)
C16—C11—P1	120.66 (15)	C42—C41—P2	118.04 (15)
C12—C11—P1	119.57 (15)	C43—C42—C41	120.1 (2)
C13—C12—C11	120.37 (19)	C43—C42—H42	119.9
C13—C12—H12	119.8	C41—C42—H42	119.9
C11—C12—H12	119.8	C44—C43—C42	119.9 (2)
C14—C13—C12	119.6 (2)	C44—C43—H43	120.1
C14—C13—H13	120.2	C42—C43—H43	120.1
C12—C13—H13	120.2	C45—C44—C43	120.3 (2)
C15—C14—C13	120.1 (2)	C45—C44—H44	119.8
C15—C14—H14	120.0	C43—C44—H44	119.8
C13—C14—H14	120.0	C44—C45—C46	120.4 (2)
C14—C15—C16	120.7 (2)	C44—C45—H45	119.8
C14—C15—H15	119.7	C46—C45—H45	119.8
C16—C15—H15	119.7	C41—C46—C45	119.7 (2)
C15—C16—C11	119.56 (19)	C41—C46—H46	120.1
C15—C16—H16	120.2	C45—C46—H46	120.1
			
C1—Ni1—P1—O1	1.53 (8)	Ni1—P1—C11—C12	178.33 (13)
P2—Ni1—P1—O1	1.95 (11)	C16—C11—C12—C13	0.1 (3)
I1—Ni1—P1—O1	−177.60 (6)	P1—C11—C12—C13	−176.83 (17)
C1—Ni1—P1—C21	115.66 (9)	C11—C12—C13—C14	−0.6 (3)
P2—Ni1—P1—C21	116.08 (11)	C12—C13—C14—C15	0.9 (4)
I1—Ni1—P1—C21	−63.48 (8)	C13—C14—C15—C16	−0.6 (4)
C1—Ni1—P1—C11	−113.72 (9)	C14—C15—C16—C11	0.1 (4)
P2—Ni1—P1—C11	−113.30 (11)	C12—C11—C16—C15	0.2 (3)
I1—Ni1—P1—C11	67.14 (7)	P1—C11—C16—C15	177.08 (17)
C1—Ni1—P2—O2	0.95 (8)	O1—P1—C21—C26	−159.66 (16)
P1—Ni1—P2—O2	0.53 (11)	C11—P1—C21—C26	−53.15 (18)
I1—Ni1—P2—O2	−179.91 (6)	Ni1—P1—C21—C26	83.46 (16)
C1—Ni1—P2—C41	115.66 (9)	O1—P1—C21—C22	23.79 (19)
P1—Ni1—P2—C41	115.25 (11)	C11—P1—C21—C22	130.30 (18)
I1—Ni1—P2—C41	−65.20 (8)	Ni1—P1—C21—C22	−93.08 (18)
C1—Ni1—P2—C31	−112.50 (9)	C26—C21—C22—C23	0.0 (3)
P1—Ni1—P2—C31	−112.92 (11)	P1—C21—C22—C23	176.56 (18)
I1—Ni1—P2—C31	66.63 (7)	C21—C22—C23—C24	−0.1 (4)
C21—P1—O1—C2	−129.06 (13)	C22—C23—C24—C25	0.0 (4)
C11—P1—O1—C2	122.75 (13)	C23—C24—C25—C26	0.2 (4)
Ni1—P1—O1—C2	−3.06 (14)	C22—C21—C26—C25	0.2 (3)
C41—P2—O2—C6	−127.56 (13)	P1—C21—C26—C25	−176.37 (16)
C31—P2—O2—C6	124.90 (13)	C24—C25—C26—C21	−0.4 (3)
Ni1—P2—O2—C6	0.88 (13)	O2—P2—C31—C36	156.25 (16)
P1—Ni1—C1—C6	176.95 (16)	C41—P2—C31—C36	51.53 (18)
P2—Ni1—C1—C6	−2.93 (15)	Ni1—P2—C31—C36	−87.80 (16)
P1—Ni1—C1—C2	0.18 (14)	O2—P2—C31—C32	−28.0 (2)
P2—Ni1—C1—C2	−179.71 (15)	C41—P2—C31—C32	−132.69 (19)
P1—O1—C2—C3	−175.11 (15)	Ni1—P2—C31—C32	88.0 (2)
P1—O1—C2—C1	3.5 (2)	C36—C31—C32—C33	0.2 (4)
C6—C1—C2—O1	−179.27 (17)	P2—C31—C32—C33	−175.7 (2)
Ni1—C1—C2—O1	−2.3 (2)	C31—C32—C33—C34	0.3 (5)
C6—C1—C2—C3	−0.7 (3)	C32—C33—C34—C35	−0.8 (5)
Ni1—C1—C2—C3	176.24 (15)	C33—C34—C35—C36	0.8 (4)
O1—C2—C3—C4	177.74 (19)	C32—C31—C36—C35	−0.2 (3)
C1—C2—C3—C4	−0.8 (3)	P2—C31—C36—C35	175.67 (17)
C2—C3—C4—C5	0.3 (3)	C34—C35—C36—C31	−0.3 (3)
C3—C4—C5—C6	1.6 (3)	O2—P2—C41—C46	135.38 (17)
C4—C5—C6—O2	178.15 (19)	C31—P2—C41—C46	−119.19 (18)
C4—C5—C6—C1	−3.3 (3)	Ni1—P2—C41—C46	17.9 (2)
P2—O2—C6—C5	175.50 (15)	O2—P2—C41—C42	−42.10 (17)
P2—O2—C6—C1	−3.1 (2)	C31—P2—C41—C42	63.33 (17)
C2—C1—C6—C5	2.8 (3)	Ni1—P2—C41—C42	−159.56 (13)
Ni1—C1—C6—C5	−174.11 (16)	C46—C41—C42—C43	0.5 (3)
C2—C1—C6—O2	−178.60 (16)	P2—C41—C42—C43	178.06 (17)
Ni1—C1—C6—O2	4.4 (2)	C41—C42—C43—C44	0.2 (3)
O1—P1—C11—C16	−115.96 (17)	C42—C43—C44—C45	−0.5 (4)
C21—P1—C11—C16	138.43 (17)	C43—C44—C45—C46	0.0 (4)
Ni1—P1—C11—C16	1.43 (19)	C42—C41—C46—C45	−1.0 (3)
O1—P1—C11—C12	60.93 (17)	P2—C41—C46—C45	−178.41 (19)
C21—P1—C11—C12	−44.68 (18)	C44—C45—C46—C41	0.7 (4)
